# Genetic Variation of Aquaporin‐4 Associates with Cognition, Brain Volume, and Dementia Risk in a Large Community Cohort

**DOI:** 10.1002/alz70860_097453

**Published:** 2025-12-23

**Authors:** Emma Louise Palatsides, Dibya Himali, Stephanie Yiallourou, Andrée‐Ann Baril, Qiong Yang, Charles S. DeCarli, Alexa S Beiser, Sudha Seshadri, Marina G. Cavuoto, Jayandra Jung Himali, Matthew P. Pase

**Affiliations:** ^1^ School of Psychological Sciences & Turner Institute for Brain and Mental Health, Monash University, Clayton, VIC, Australia; ^2^ Boston University School of Medicine, Boston, MA, USA; ^3^ School of Psychological Sciences & Turner Institute for Brain and Mental Health, Monash University, Melbourne, VIC, Australia; ^4^ Center for Advanced Research in Sleep Medicine, Hôpital du Sacré‐Coeur de Montréal, CIUSSS‐NIM, Montreal, QC, Canada; ^5^ Université de Montréal, Montreal, QC, Canada; ^6^ Department of Biostatistics, Boston University School of Public Health, Boston, MA, USA; ^7^ Department of Neurology & Imaging of Dementia and Aging Laboratory, University of California, Davis, Davis, CA, USA; ^8^ Department of Neurology, Boston University Chobanian & Avedisian School of Medicine, Boston, MA, USA; ^9^ Glenn Biggs Institute for Alzheimer's & Neurodegenerative Diseases, San Antonio, TX, USA; ^10^ University of Texas Health San Antonio, San Antonio, TX, USA; ^11^ National Ageing Research Institute, Melbourne, VIC, Australia; ^12^ Monash University, Clayton, VIC, Australia

## Abstract

**Background:**

The aquaporin‐4 (AQP4) water channel plays an integral role in removing waste from the brain, including β‐amyloid and tau proteins. Currently, little is known about whether genetic differences in the *AQP4* gene are involved in the development of dementia. We aimed to study the association between genetic variation at an *AQP4* haplotype with cognition, brain volumes, and incident dementia.

**Method:**

Participants were from the community‐based Framingham Heart Study (FHS) first, second, and third‐generation cohorts. All participants were dementia‐free at the time of cognitive assessment, brain MRI, and the commencement of dementia follow‐up. Linear regression models were conducted to examine the relationship between genetic variation of *AQP4* with cognition and brain volumes for participants aged 45 years and older. Cox proportional hazards regression models were conducted to examine the association between genetic variation of *AQP4* with incident dementia beginning from age 65 ± 2.5, 70 ± 2.5, and 75 ± 2.5 years at baseline, with a maximum of a 20‐year follow‐up. Analyses were adjusted for age, sex, cohort, *APOE* (ε4 carrier), age squared (for cognitive and MRI outcomes), and education (for cognitive outcomes).

**Results:**

3847 participants had a cognitive assessment; 3332 had MRI. Carriage of the minor allele at the *AQP4* haplotype was associated with better verbal episodic memory (*β*[95% CI], 0.325 [0.10, 0.55], *p* = 0.004) and larger hippocampal volumes (*β*[95% CI], 0.004 [0.001, 0.007], *p* = 0.009) compared to homozygotes for the major allele. Carrying at least one copy of the minor allele was associated with lower incident dementia risk across each age band. This reached borderline significance from age 75 onwards, with heterozygotes displaying a 32% lower risk of incident AD dementia compared to homozygotes for the major allele (HR = 0.68, 95% CI [0.47, 1.00], *p* = 0.049, *n* = 551, incident cases = 35).

**Conclusion:**

Carrying the minor allele at the *AQP4* haplotype was associated with better episodic memory, larger hippocampal volumes, and trends for lower dementia risk. Further research exploring the role of AQP4 may identify additional mechanism contributing to dementia onset and progression.

